# Unmasking of Gitelman Syndrome during Pregnancy in an Adolescent with Thyrotoxic Crisis

**DOI:** 10.3390/pediatric13040075

**Published:** 2021-12-01

**Authors:** Ratna Acharya, Kiran Upadhyay

**Affiliations:** 1 Division of General Pediatrics, Department of Pediatrics, University of Florida, Gainesville, FL 32610, USA; racharya@ufl.edu; 2 Division of Pediatric Nephrology, Department of Pediatrics, University of Florida, Gainesville, FL 32610, USA

**Keywords:** Gitelman, hypokalemia, thyrotoxicosis, pregnancy

## Abstract

Background. Gitelman syndrome (GS) is an inherited salt-losing renal tubulopathy characterized by hypokalemic metabolic alkalosis with hypomagnesemia and hypocalciuria. Patients can be asymptomatic until late adolescence or adulthood, and hence may be discovered incidentally during presentation with other illnesses. GS has been described in association with thyroid disorders and should be considered in patients with hyperthyroidism and persistent hypokalemia, especially in those with associated hypomagnesemia and hypocalciuria. Case summary. In this report, we describe an 18-year-old female who presented with hyperemesis gravidarum and thyrotoxicosis, and was incidentally found to have GS, confirmed by the sequence analysis of *SLC12A3.* Conclusions. Thyroid dysfunctions, such as hypothyroidism, thyrotoxicosis, and thyroid nodules, may develop during pregnancy. A structural homology between the beta-human chorionic gonadotropin and thyroid stimulating hormone molecules, as well as their receptors is probably the basis for the transient thyrotoxicosis crisis during pregnancy. Since hyperemesis in pregnancy can also lead to hypokalemia and alkalosis, a high index of suspicion for GS during pregnancy is required for timely diagnosis and management.

## 1. Introduction

Gitelman syndrome (GS) is an autosomal recessive renal tubulopathy characterized by hypokalemia, metabolic alkalosis, hypomagnesemia, and hypocalciuria [1]. GS is caused by a loss-of-function mutation in the solute carrier family 12, member 3 (*SLC12A3*) gene which encodes a thiazide-sensitive sodium chloride cotransporter in the distal convoluted tubule [2]. The increased sodium delivery in the cortical collecting duct leads to increased sodium reabsorption from the epithelial sodium channel, and simultaneously increased potassium and magnesium wasting. If severe, hypokalemia can cause periodic paralysis (PP) [3]. Common clinical features include transient periods of muscle weakness and tetany that may be accompanied by abdominal pains, vomiting, and fever. 

PP can also occur secondary to thyroid disorders. Thyrotoxic PP is a sporadic form of hypokalemic PP that occurs in association with hyperthyroidism [4]. This is in contrast with the familial hypokalemic PP, which is inherited in an autosomal dominant pattern and is due to mutations in *ACNA1S* or *SCN4A* gene which alter the structure and function of calcium or sodium channels [5]. Our patient did not have family history of PP. Thyrotoxic PP manifests as recurrent episodic muscle weakness with hypokalemia and hyperthyroidism. It mostly has been described in Asian men and is caused by hyperstimulation of Na^+^/K^+^-ATPase channel due to high thyroxine level that results in the rapid shift of potassium into the cells [6]. Concurrent GS and thyrotoxic PP has been described, both in children and adults [7,8,9]. Imashuku et al. described a 16-year-old Asian male with thyrotoxic PP with a concurrent GS secondary to a missense homozygous mutation of *SLC12A3* [7].

Acute kidney injury (AKI) and end stage renal disease (ESRD) have been described with GS [10,11]. Nishihara et al. described an adult male with AKI secondary to hypokalemic rhabdomyolysis in association with GS [10]. Lee et al. described a 27-year-old male with GS with ESRD secondary to persistent vomiting [11]. 

In this report, we describe an 18-year-old adolescent who was incidentally found to have GS during work-up for severe hypokalemia and hyperthyroidism during the early second trimester of pregnancy.

## 2. Case Presentation

An 18-year-old adolescent female was evaluated at 15 weeks’ gestation for history of persistent emesis for four weeks, extreme fatigue and red colored urine for one day. There was no history of fever, diarrhea, abdominal pain, or urinary tract infection. 

Past medical history was unremarkable for diabetes mellitus, rheumatoid arthritis, or other autoimmune diseases. She was not known to have hyperthyroidism in the past. Pregnancy had been uneventful except for persistent episodes of emesis for past one month. There was no prior history of mood symptoms or altered mental status. There was no history of heat intolerance, diarrhea, or weight loss. There was no recent history of usage of corticosteroid, loop or thiazide diuretic, strenuous exercise, vaginal bleeding, vaginal discharge of grape-like vesicles, seizures, alcohol intake, and carbohydrate load. Family history was notable only for type 2 diabetes in the father. There was no known family history of autoimmune diseases, thyroid disorders, hypokalemia, and periodic paralysis. There was no consanguinity.

Vital signs upon presentation showed blood pressure (BP) 142/87 mm Hg, pulse 120 beats per minute, oral temperature 38.9 °C (102.02 °F), respiratory rate 18 per minute, and oxygen saturation of 97%. Her height was 172.7 cm and weight was 109 kg (240 lb 4.8 oz). Physical examination was remarkable for an overweight female who was awake but slow to respond. There was no goiter and ophthalmopathy. There was mild scleral icterus. There was no tremor or pretibial edema.

Serum beta-human chorionic gonadotropin (hCG) level was 246,000 mIU/mL and sonogram confirmed the presence of a single viable intrauterine fetus of approximately 15 weeks gestation with expected uterine size. Renal function test showed sodium 127 mmol/L, potassium 2.5 mmol/L, bicarbonate 33 mmol/L, blood urea nitrogen (BUN) 123 mg/dL, serum creatinine 4.2 mg/dL (MDRD estimated glomerular filtration rate (eGFR) 17 mL/min/1.73 m^2^), calcium 9.5 mg/dL, phosphorus 2.7 mg/dL and magnesium of 1 mg/dL. Liver function test showed aspartate aminotransferase 140 IU/L, alanine aminotransferase 203 IU/L, total bilirubin 2.9 mg/dL, ammonia 55 µmol/L, and serum albumin 3.1 gm/dl. Renal function test two months prior to pregnancy showed normal serum creatinine of 0.8 mg/dL (MDRD eGFR 113 mL/min/1.73 m^2^) and normal electrolytes (serum sodium 138 mmol/L, potassium 3.9 mmol/L, bicarbonate 24 mmol/L, calcium 9.4 mg/dL, phosphorus 3.9 mg/dL, and magnesium was not available) Serum creatine kinase was normal at 121 U/L (normal 0–180 U/L). Urine sodium was <10 mmol/L, urine creatinine 84 mg/dL, urine potassium 28 mmol/L and urine osmolality was 344 mOsm/kg. Fractional excretion of sodium was 0.4%. Urinalysis showed 1+ proteinuria, no microscopic hematuria, 5 white cells per high power field, no ketonuria, pH 5, specific gravity of 1.015, and negative nitrites and leukocytes. Plasma renin activity was 14 ng/mL/h (normal 0.5–4 ng/mL/h, upright) and serum aldosterone was 13 ng/dL (normal 4–31 ng/dL, upright). Blood and urine cultures were negative. Other extensive investigations for infectious etiologies were also negative. Urine for Chlamydia and Neisseria was negative. SARS-CoV-2 DNA PCR from the nasopharyngeal swab was negative. Other labs were notable for undetectable thyroid stimulating hormone (TSH) level < 0.030 mIU/L (reference 0.4–5 mIU/L), total T3 238 ng/dL (reference 87–178 ng/dL), and free T4 > 6.99 ng/dL (reference 0.6–1.2 ng/dL). Computed tomography of the head showed no acute intracranial abnormalities. 

Initial management included administration of intravenous fluids (IVF), propranolol, potassium iodide, propylthiouracil (PTU), and hydrocortisone. A presumptive diagnosis of hyperemesis gravidarum with thyroid storm and AKI secondary to persistent emesis was made. She met criteria for thyroid storm due to elevated serum free T4 and total T3 with undetected TSH, along with pyrexia, tachycardia, icterus, and altered mental status. Her clinical condition improved somewhat after receiving PTU, propranolol, and hydrocortisone as heart rate, blood pressure, and free T4 began to decrease. Her mental status improved and she became more alert and engaged in conversation. Given tachycardia and hypertension, she was started on propranolol 60 mg every 4 hours which was later converted to 160 mg daily. 

Other thyroid studies showed thyroid peroxidase antibody 0.3 IU/mL (reference < 9 IU/mL), TSH receptor antibody (TRAb) < 0.90 (reference < 1.75 IU/L), and undetected thyroid stimulating immunoglobulin (TSI) of < 0.10 (reference ≤ 0.54 IU/L). Given negative TRAb, Graves’ disease was highly unlikely. Serum free T4 normalized in ten days, TSH in two weeks and total T3 in five days. Given pregnancy, further modalities including radioactive iodine uptake study were not pursued, given risk of fetal hypothyroidism, mental retardation, and increased risk of malignancy. Partial molar pregnancy was initially considered due to presentation of thyroid storm, however, the beta-hCG level was trending down and the patient did not show any signs of pre-eclampsia. Thyroid sonogram showed normal sized thyroid with homogenous thyroid texture and without increased vascularity, making Grave’s disease unlikely. Investigations for AKI included renal sonogram which showed right kidney measuring 12.8 cm in length and left kidney measuring 12.6 cm in length with bilateral normal cortical echogenicity without evidence of hydronephrosis or nephrocalcinosis. Hepatic sonogram showed gallbladder sludge. A chest X-ray was normal without pulmonary edema or cardiomegaly. Electrocardiogram showed normal Q-T interval. Echocardiogram was normal with ejection fraction of 65%. Cardiac enzymes were normal. 

PTU was later changed to methimazole as she was no longer in the first trimester of pregnancy and as she demonstrated signs of liver insufficiency. Anti-emetics and IVF were discontinued after few days as the patient was able to tolerate well by mouth. Hydrocortisone was discontinued after two days. Subsequently, propranolol was discontinued along with methimazole, as the beta-hCG mediated hyperemesis was thought to be the likely etiology of thyroid symptoms. Her vital signs, including BPs, remained stable and her thyroid levels returned to normal without anti-thyroid drugs. Serum beta-hCG at discharge was 175,557 mIU/mL. Given decreasing level of hCG without other signs or symptoms of gestational trophoblastic disease, molar pregnancy was unlikely. She was discharged without any thyroid medications.

During the hospitalization, her serum potassium remained around 2.4–2.9 meq/L along with serum bicarbonate 30–38 meq/L despite resolution of emesis. Serum magnesium remained low, as well as with values ranging from 1–1.4 mg/dL. Twenty-four hour urine collection showed a urine calcium of 0.04 mg/kg/day. Random urine calcium to creatinine ratio was also low at 0.005. She required multiple intravenous potassium and magnesium supplements. Discharge serum potassium and magnesium was 3.5 meq/L and 1.2 mg/dL, respectively. She was discharged on magnesium oxide 400 mg daily and potassium chloride 10 meq twice daily. BUN and serum creatinine slowly improved with intravenous hydration. Kidney biopsy was not performed and renal replacement therapy was not required. Discharge BUN and serum creatinine was 15 mg/dL and 0.97 mg/dL (MDRD eGFR 90 mL/min/1.73 m^2^), respectively. Due to the persistent hypokalemia, alkalosis, hypomagnesemia and hypocalciuria despite resolution of emesis, a genetic testing by next generation sequence analysis was done which showed that the patient was homozygous in the *SLCA123* (NM_001126108.2) gene for a known sequence variant designated c.2581C>T (p.Arg861Cys) (Prevention Genetics, Marshfield, WI, USA). No further in vitro functional studies were performed, hence the exact functional implication of this variant was unknown. However, to determine the pathogenicity of this variant, in silico tests were performed using SIFT, PolyPhen-2, FATHMM and MutationTaster. The resulting prediction utilizing these in silico tools was “Pathogenic”. At 37 weeks gestation, a healthy infant of 3.5 kilograms was born vaginally without any perinatal complications. The serum creatinine remained stable at 0.7 mg/dL after delivery (MDRD eGFR 132 mL/min/1.73 m^2^).

## 3. Discussion

The etiologies of hypokalemia include diarrhea, alcohol intake, intrinsic renal tubular transport disorders, such as Bartter and Gitelman syndromes, and tubular injuries from nephrotoxic drugs, including aminoglycosides, amphotericin B, and cisplatin. Other disorders, such as familial hypokalemic PP, thyrotoxic PP, and renal tubular acidosis, can present with hypokalemia as well. During pregnancy, hypokalemia can also occur from hyperemesis gravidarum. The patient in this report did have hyperemesis but the metabolic abnormalities persisted after resolution of emesis and the sequence analysis of the *SLC12A3* gene confirmed GS. Hence, the constellation of hypokalemia, hypomagnesemia, hypocalciuria, alkalosis, mild activation of renin-angiotensin system (RAS), and mutation in *SLC12A3* gene was confirmatory of GS. The mild activation of the RAS was most likely the appropriate regulatory response to dehydration secondary to renal salt-wasting. Additionally, thyroid hormones are known to influence the synthesis and secretion of renin by juxtaglomerular (JG) cells. T3 has been shown to increase renin secretion, renin content, and renin mRNA in rat JG cell cultures [12]. In our patient, the concomitant presence of severe transient hyperthyroidism made thyrotoxic PP another possibility. However, given that the TSI was normal and patient had no prior history of hyperthyroidism, the transient elevation of thyroid hormones and suppression of TSH was thought to be secondary to hCG-related. 

Although the patients with GS are mostly asymptomatic, episodes of muscle weakness and tetany can occur. The prevalence is 1 in 40,000 and the diagnosis is usually made in late adolescence or early adulthood, sometimes incidentally during presentation of other conditions which are known to cause hypokalemia. Jalalzadeh et al. reported a diagnosis of GS in a patient presenting with diabetic ketoacidosis [13]. Zhou et al. reported Graves’ disease in a patient with GS [9]. Although pregnancy with GS has been reported with varying outcomes [14,15], a new diagnosis of GS during pregnancy has not been reported. Potassium and magnesium loss can occur in pregnancy due to increased renal tubular loss, partly due to activation of renin-angiotensin system [16]. The presence of hyperemesis and fetal demands can also worsen hypokalemia in patients with GS during pregnancy. However, pregnancy typically is associated with retention of potassium, which is thought to be mediated by progesterone due to its mineralocorticoid effect which, in turn, can prevent excess kaliuresis [17,18]. It needs to be studied whether this protective mechanism is maintained in pregnant patients with underlying GS as the loss of urinary electrolytes can result in severe hypokalemia and hypomagnesemia during pregnancy in these patients [19]. Hence, a high index of suspicion for GS is necessary in pregnant patients with severe or refractory hypokalemia and hypomagnesemia. The outcome of pregnancy in patients with GS is favorable as long as electrolyte supplementation is adequately managed. However, increased risks of miscarriage, oligohydramnios and intrauterine growth retardation have been reported [20,21].

Gestational thyrotoxicosis (GT) is a transient form of hyperthyroidism caused by excessive stimulation of thyroid gland by hCG and usually limited to the first 12–16 weeks of pregnancy [22]. It is known to affect mostly women with hyperemesis gravidarum, multiple gestation, molar pregnancy, and choriocarcinoma [23]. The thyroid stimulating effect of hCG is thought to be due to the significant homology between the beta-subunits of hCG and TSH. As hCG secretion declines, serum free T4 and T3 concentrations decline and serum TSH concentrations rise slightly to or within the normal range. Hence, it is necessary to understand the thyroid physiology during pregnancy that can cause altered thyroid function tests. Additionally, it is recommended that during normal pregnancy, these tests should be interpreted using population based, trimester-specific TSH and T4 reference ranges for pregnant women [24]. Patients usually have a suppressed (<0.1 mU/L) or undetectable (<0.01 mU/L) serum TSH value and a free T4 and/or free T3 (or total T4 and/or total T3) measurement that exceeds the normal range for pregnancy. GT resolves on its own mostly by early second trimester and no anti-thyroid drugs are indicated [22]. Another important cause of persistent hyperthyroidism in pregnancy is Graves’ disease which usually manifests with overt signs and symptoms of hyperthyroidism, diffuse non-tender goiter with or without bruit, various degrees of opthalmopathy along with a positive serum thyroid peroxidase antibody and a positive serum TSH receptor antibody test [25]. Anti-thyroid drugs are necessary to treat Graves’ disease. Other causes of hyperthyroidism in pregnancy are toxic adenoma, toxic multinodular goiter, and subacute thyroiditis [25].

Thyroid storm, also referred to as thyrotoxic crisis, is an acute, life-threatening, decompensated, hypermetabolic state induced by excessive release of thyroid hormones in patients with thyrotoxicosis. It is typically seen in the setting of Grave’s disease and the clinical presentation includes fever, tachycardia, hypertension, neurological, and gastrointestinal abnormalities; heart failure, delirium, and seizures can occur [26]. Superimposed insults, such as infection, trauma, surgery, myocardial infarction, diabetic ketoacidosis, pregnancy, and parturition, have been known to trigger the thyroid storm. Additionally, hyperemesis in pregnancy has been reported as a cause of thyroid storm in the first and second trimesters of pregnancy [27]. Return of thyroid levels to normal with resolution of emesis and without anti-thyroid agents suggests the role of beta-hCG in the GT, as seen in our patient. Beta-hCG is known to share some structural homology with TSH [28]. Our patient had markedly elevated serum beta-hCG level, and cross-reactivity with TSH based on identical alpha subunit has been described. An inverse relationship between TSH and beta-hCG at about 10–12 weeks of pregnancy, the time of peak hCG levels, has been well described [29]. There is also a two-three fold estrogen-mediated increase in circulating levels of thyroid-binding globulin (TBG) during pregnancy [30]. TBG, which is one of the proteins which transport thyroid hormone in the blood with high affinity for thyroxine (T4) increases in serum a few weeks after conception and reaches a plateau during the mid-gestational period. The mechanism for this increase in TBG involves both increased hepatic synthesis of TBG and estrogen-mediated perpetuation in sialylation of TBG that increases the half-life from 15 min to three days to fully sialylated TBG [31].

Treatment of thyroid storm is typically with a thioamide (methimazole or PTU) and iodine. Iodine needs to be given after the administration of a thioamide to prevent worsening of thyroid storm as iodine could instead lead to the Jod–Basedow phenomenon. Glucocorticoids can also be used in thyroid storm to potentially decrease T4 to T3 conversion, improve hemodynamics and potentially suppress the autoimmune process. Beta blockade is important for cardiovascular symptoms but should be used cautiously if heart failure is present. Propranolol is typically preferred in pregnancy. 

Methimazole is typically avoided in the first trimester due to the risk of esophageal or choanal atresia and aplasia cutis but is safe in the second and third trimester. PTU has a more rapid onset of action and, therefore, can be useful in thyroid storm and also decreases T4 to T3 conversion but has a higher incidence of hepatotoxicity. Both medications can rarely lead to agranulocytosis. 

Hypomagnesemia is associated with hypokalemia. Concomitant magnesium deficiency aggravates hypokalemia and renders it refractory to treatment by potassium supplementation. Impairment of Na-K-ATPase caused by magnesium deficiency contributes to potassium wasting [32]. A decrease in cellular uptake of potassium, in patients with gastrointestinal loss, such as vomiting, may lead to severe potassium wasting and hypokalemia. Vomiting itself is associated with decreased urine potassium loss but, in someone with associated GS and hypomagnesemia, it may lead to increased urinary potassium loss. Pregnant patients may be more susceptible to hypokalemia and hypomagnesemia due to increased renal tubular loss from activation of renin angiotensin system.

## 4. Conclusions

Here, we report a case of incidental new diagnosis of GS in a patient with thyroid storm who presented with hyperemesis gravidarum. GS should be considered in patients with hyperthyroidism and persistent hypokalemia, especially in those with hypomagnesemia and hypocalciuria.

## Data Availability

No new data were created or analyzed in this study. Data sharing is not applicable to this article.
